# PLCD1-Induced DNA Damage Inhibits the Tumor Growth via Downregulating CDKs in Chondrosarcoma

**DOI:** 10.1155/2022/4488640

**Published:** 2022-07-04

**Authors:** Jiakang Shen, Chen Yu, Zhuoying Wang, Haoran Mu, Zhengdong Cai

**Affiliations:** ^1^Shanghai General Hospital of Nanjing Medical University, Shanghai, China; ^2^Department of Orthopaedics, Shanghai Bone Tumor Institute, Shanghai General Hospital, Shanghai Jiao Tong University School of Medicine, Shanghai 200080, China; ^3^Sir Run Run Hospital, Nanjing Medical University, Nanjing 211100, China

## Abstract

**Purpose:**

Typical genes for the treatment and diagnosis of high-grade chondrosarcoma are still in need. Our study aimed to explore the PLCD1 function in chondrosarcoma for further treatment.

**Materials and Methods:**

Our study collected the information of 49 patients in our department. The PLCD1 expression in our cohort was detected and was compared with the TCGA database. PLCD1 knockdown and overexpression cell lines were established stably. Cell viability assay and colony formation assay were performed for cell proliferation. Flow cytometry analysis was performed for cell cycle and apoptosis. Western blotting was performed for PLCD1-related protein expression. Animal xenografts were established to verify the effect of PLCD1 in high-grade chondrosarcoma.

**Results:**

Compared with the TCGA database, the relation between PLCD1 expression and the malignancy of chondrosarcoma was demonstrated. A lower PLCD1 expression was detected mainly in high-grade chondrosarcoma. PLCD1 overexpression in high-grade chondrosarcoma suppressed CDKs/cyclins and induced DNA damage causing cell cycle blocking and apoptosis. Antitumor effect of PLCD1 overexpression was verified in vivo.

**Conclusion:**

Lower PLCD1 was expressed in high-grade chondrosarcoma. Overexpressed PLCD1-induced DNA damage caused cell cycle blocking and apoptosis in vitro and in vivo. PLCD1 could be a novel target in high-grade chondrosarcoma for further drug development.

## 1. Introduction

Chondrosarcoma (CS) is one of the most primary bone tumors, and development of novel treatment is still in need for the poor prognosis of high-grade chondrosarcoma [1–3]. Hallmarks of high-grade chondrosarcoma are enrichment in the cell proliferation pathway [2, 4], and the related gene is considered to be a novel therapeutic target.

Phospholipase C (PLC) is an important enzyme in the phosphoinositide metabolism [5–7], and PLCD1, a subgroup in the PLC isozyme family, is involved in tumorigenesis and cytoskeleton transformation [8–10]. PLC enzyme hydrolyzes phosphatidylinositol-4,5-bisphosphate to generate inositol-1,4,5-trisphosphate and 1,2-diacylglycerol in the system of the phosphoinositide metabolism.

Recent research has demonstrated that PLCD1 shows obvious antitumor effects such as decreasing cell proliferation in breast cancer, esophageal squamous cell carcinoma, and colon-rectal cancer [9–15]. Antiproliferation of PLCD1 reminds us that it may be a novel target for the treatment of high-grade chondrosarcoma, and our study aims to explore the function of PLCD1 protein in high-grade chondrosarcoma.

## 2. Materials and Methods

### 2.1. Patient Cohort

The patients admitted to our department from 2009 to 2015 and diagnosed with chondrosarcoma were included in our study. The tumor tissues of the patients were fixed in neutral buffered formalin and processed routinely with paraffin embedding. Three pathologists examined hematoxylin and eosin-stained glass slides from each tumor sample, and 1 paraffin block from each tumor sample was selected for immunohistochemistry (IHC). The known clinical-histological features and IHC results of patients have also been collected. The present research was approved by the Institutional Research Ethics Committee of Shanghai General Hospital, and informed consent was obtained from all patients (2008KY018).

## 3. Reagents and Chondrosarcoma Cell Culture

### 3.1. Reagents

Monoclonal antibodies (anti-PCNA, anti-CDK2, anti-CDK4, anti-CDK6, anti-cyclin D1, anti-c-myc, and anti-GAPDH) were from Cell Signaling Technology (Beverly, MA, USA). Rabbit monoclonal antibody (anti-PLCD1 (ab134936)) was obtained from Abcam Technology (Abcam, Cambridge, MA, USA). Quinoline-Val-Asp-difluorophenoxymethylketone (Q-VD-Oph, S7311) was purchased from Selleck Chemicals (Houston, USA). Homologous Recombination (HR) DNA Repair Antibody Sampler Kit (#99891) was purchased from Cell Signaling Technology (Beverly, MA, USA).

### 3.2. Chondrosarcoma Cell Lines and Cell Culture

Human chondrosarcoma cell lines HCS-2/8 [16] and SW1353 [17] and human osteoblast cell line hFOB1.19 were mainly used. SW1353 was from American type culture collection (ATCC HTB-94), HCS-2/8 was from Dr. J Block's laboratory (Rush Medical College, Chicago, IL, USA), and hFOB1.19 was from American type culture collection (ATCC CRL-11372). HEK-293 T cell line was from ATCC (ACS-4500). SW1353 was cultured with L-15 and HCS-2/8 was maintained in DMEM supplemented with 10% FBS and 1% antibiotics. All cell lines used were tested for mycoplasma in routine.

### 3.3. Immunohistochemistry

IHC assays were performed as per previous reports [18]. Paraffin sections were made to react with rabbit polyclonal anti-PLCD1 antibodies (1: 200 dilution). Sections stained with nonimmune rabbit serum (1: 200 dilution) in phosphate-buffered saline (PBS) instead of the primary antibody served as negative controls. Cells exhibiting positive staining in the cytoplasm and nucleus were counted in at least 10 representative fields (× 400 magnification), and the mean percentage of positive cells was calculated. Immunostaining was assessed by two independent pathologists blinded to clinical characteristics and outcomes.

### 3.4. Cell Proliferation Assays

Cells were seeded in 96-well plates at a density of 5000 cells per well and cultured overnight. Cell proliferation was detected by the reagent CCK-8 (Dojindo Laboratories, Kumamoto, Japan) according to the manufacturer's instructions. The absorbance at 450 nm was measured using a microplate reader iMark (Molecular Devices, Sunnyvale, CA, USA).

### 3.5. Stable Silenced or Overexpressing Cell Lines

Transfections were performed using Lipofectamine 3000 (Thermo Scientific) according to the manufacturer's protocols. The sequence of targeted PLCD1 was 5′-GACGGCTTCCTCATGTACTTA-3′; the control sequence was 5′-CAACAAGATGAAGAGCACCAA-3′. Cells were transfected using the plasmids in 293T. After 48 hours, the supernatant containing packaged lentivirus was harvested and used on SW1353 cells or HCS-2/8 cells for 24 hours. Puromycin was performed to screen the stable knockdown cell lines. Overexpression plasmids were purchased from Youbio Biological Technology Co., Ltd. (Changsha, China). According to the manufacturer's instruction, the stable overexpressed cell lines were established in SW1353 and HCS-2/8.

### 3.6. Western Blotting

Total protein was extracted from gathered cells with RIPA protein lysis buffer (Beyotime, Shanghai, China) on cold ice (4°C) in about 30 mins. The protein then was collected and centrifuged (12000 rpm) at 4°C for 20 min. The supernatant was collected, and the protein concentration of the lysates was measured using the Protein BCA Assay Kit (Bio-Rad, Hercules, CA, USA). For the western blotting assay, 20 *μ*g of protein mixed with 5 × SDS loading buffer was loaded per lane, separated with 10% SDS-PAGE, and transferred to a polyvinylidene fluoride (PVDF) membrane (MilliporeSigma, Burlington, USA). After blocking with 5% skimmed milk in TBST for 1 h at room temperature, the membrane was incubated at 4°C overnight with primary antibodies, and then, the membrane was washed three times for 10 min with TBST solution (TBS, 1×; Tween-20, 1:1000) and incubated for 1 h with the corresponding HRP-conjugated secondary antibodies (1:5000, Abgent, San Diego, USA). Chemiluminescent detection was performed using an ECL kit (Pierce Chemical, Rockford, IL, USA) and Bio-Rad ChemiDoc MP Imaging System (Bio-Rad, Hercules, CA, USA). All experiments were performed in triplicate.

### 3.7. Colony Formation Assay

Cells were plated evenly in six-well plates at a density of 200 cells per well. After incubation for 2 weeks, the medium was discarded, and then, the cells were washed with PBS three times. After fixation with paraformaldehyde (4%), the colonies were stained with crystal violet (0.1%) for 15 minutes. The colony number was counted manually.

### 3.8. Flow Cytometric Analysis of Cell Cycle and Apoptosis

Cell lines were plated evenly in six-well plates at a density of 5 × 10^5^ cells per well and treated according to the requirements. Then, the cells were collected and washed with ice-cold PBS three times. The cells were resuspended in the PI staining solution (C1052, Beyotime, Shanghai, China) for 30 minutes, and then, the samples' cell cycle were detected by flow cytometry (BD Biosciences, Mountain View, CA, USA). The harvested cells were resuspended in the binding buffer containing Annexin V-FITC/PI (BD Biosciences, Mountain View, CA, USA). After 15 minutes of incubation in dark at room temperature, the cells' apoptosis was effectively detected by flow cytometry.

### 3.9. Animal Model

All animal care and experimental studies were performed according to the guidelines of the Animal Investigation Committee of the Shanghai General Hospital (2018SQ038).

Male BALB/c nude mice (4–6 weeks) were maintained and bred in the animal center of Shanghai General Hospital.

HCS-2/8 cells and PLCD1 overexpression cells of HCS-2/8 were plated into the right flank of each nude mouse (1 × 10^6^ cells). After 1 week, body weight and tumor sizes were measured every three days. Tumor volume was calculated using the following equation: tumor volume = (length × width^2^)/2.

### 3.10. TUNEL Assay

The tumor tissue apoptosis was detected using the TUNEL Assay Kit (Beyotime) as per manufacturer's instruction. In brief, paraffin-embedded tumor sections were deparaffinized using xylene and rehydrated ethanol and treated with proteinase K for antigen retrieval. After washing three times with PBS, the tumor sections were treated with a TUNEL detection mixture in a humidified chamber at 37°C for 1 hour. After washing with PBS, the sections were treated with DAPI staining buffer, and chosen fields at random with apoptotic cells were observed using a fluorescence microscope (Leica, Wetzlar, Germany).

### 3.11. Bioinformation Analysis

PLCD1 expression in Pan-cancer database The Cancer Genome Atlas (TCGA) (https://www.cancer.gov/about-nci/organization/ccg/research/structural-genomics/tcga) and pan-cancer cell line database Cancer Cell Line Encyclopedia (CCLE) (https://sites.broadinstitute.org/ccle/) was analyzed in our study, and the survival rate according to PLCD1 expression was analyzed by online tool GEPIA (http://gepia.cancer-pku.cn/detail.php?gene=PLCD1).

### 3.12. Statistical Analysis

All data are presented as the mean ± standard deviation (SD) from three independent experiments at least. Fisher's exact test, unpaired Student's *t*-test, and one-way ANOVA were used to compare differences between the control and treatment groups. *P* values < 0.01(*∗*) were considered significant.

## 4. Results

### 4.1. PLCD1 Expression Predicted the Chondrosarcoma Malignancy

**Figure 1:**
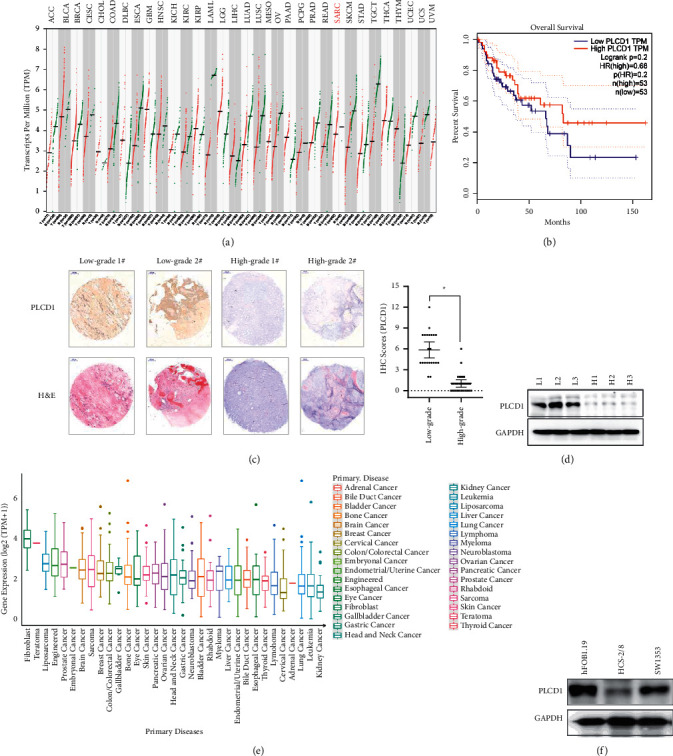
PLCD1 expression predicted the chondrosarcoma malignance. (a) Pan-cancer PLCD1 expression in the TCGA database. (b) Overall survival of sarcoma cases in TCGA by PLCD1 expression. (c) Immunohistochemistry revealed higher PLCD1 expression in malignant chondrosarcomas (high grade). (d) Western blotting of PLCD1 protein expression grouped by malignance. (e) Pan-cancer cell line PLCD1 expression in the CCLE database. (f) Western blotting of PLCD1 protein expression in chondrosarcoma cell lines.

Histological classification of chondrosarcoma is still not clear in that related biomarker is in need to distinguish more malignant subgroup from others. Compared with the TCGA database (Figures 1(a) and 1(b)), our immunohistochemical results of chondrosarcoma tissue microarray (49 samples) revealed the relation between PLCD1 expression and malignance of chondrosarcoma, compared with H&E (Figure 1(c)). A lower PLCD1 expression was mainly found in high-grade chondrosarcoma and was verified by western blotting (Figure 1(d)). We further detected the PLCD1 expression in the CCLE database (Figure 1(e)) and chondrosarcoma cell lines (HCS-2/8 and SW1353) (Figure 1(f)). A higher PLCD1 expression in HCS-2/8 indicated it might be more malignant.

### 4.2. PLCD1 Expression Regulated Chondrosarcoma Cell Proliferation In Vitro

To further reveal the relation between PLCD1 expression and malignance, the stable PLCD1 knockdown/overexpression cell lines were established to verify PLCD1 function in chondrosarcoma cell lines.

### 4.3. Cell Proliferation Was Promoted by PLCD1 knockdown In Vitro

**Figure 2:**
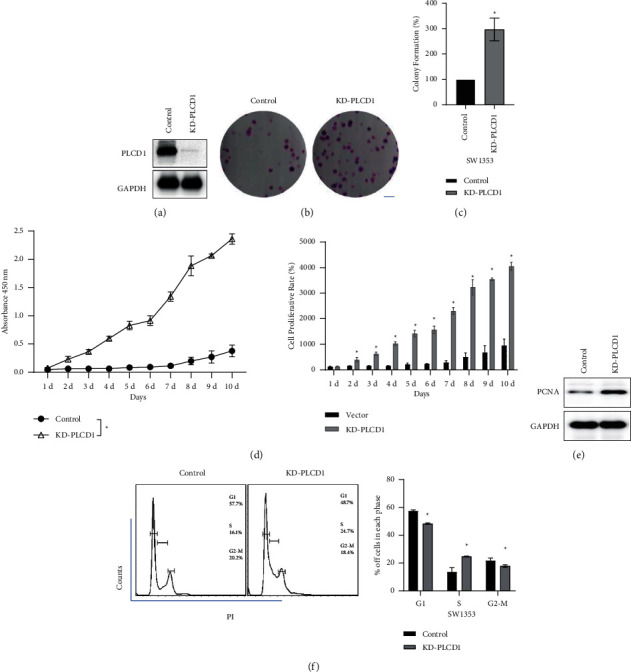
PLCD1 knockdown promoted cell proliferation in vitro. (a) Establishment of the KD-PLCD1 SW1353 cell line. (b, c) Colony formation of the KD-PLCD1 cell line. (d) CCK-8 assay kit revealed a downregulation of cell proliferation by PLCD1 knockdown. (e) Western blotting revealed an upregulation of PCNA expression by PLCD knockdown. (f) Flow cytometry analysis revealed cell cycle blocking by PLCD knockdown.

PLCD1 knockdown cell line (KD-PLCD1) was established based on the SW1353 cell line (Figure 2(a)). The results of the colony formation assay (Figures 2(b) and 2(c)) demonstrated that colonies of KD-PLCD1 were significantly increased and the colony formation rate approximately tripled from that of the control group. CCK-8 assay revealed the cell viability of KD-PLCD1 increased compared with the control group (Figure 2(d)). Western blotting verified the expression of proliferating cell nuclear antigen (PCNA), a marker protein of cell proliferation, enhanced in the PLCD1 KD group (Figure 2(e)). Flow cytometry assay of the cell cycle was carried out to further investigate the effect of cell proliferation on PLCD1 knockdown (Figure 2(f)). The KD-PLCD1 group contained more S phase but fewer G1 phase cells than the control group. Our results demonstrated that PLCD1 knockdown in chondrosarcoma cells in vitro promoted cell proliferation by enhancing the cell cycle.

### 4.4. Cell Proliferation Was Inhibited by PLCD1 Overexpression

**Figure 3:**
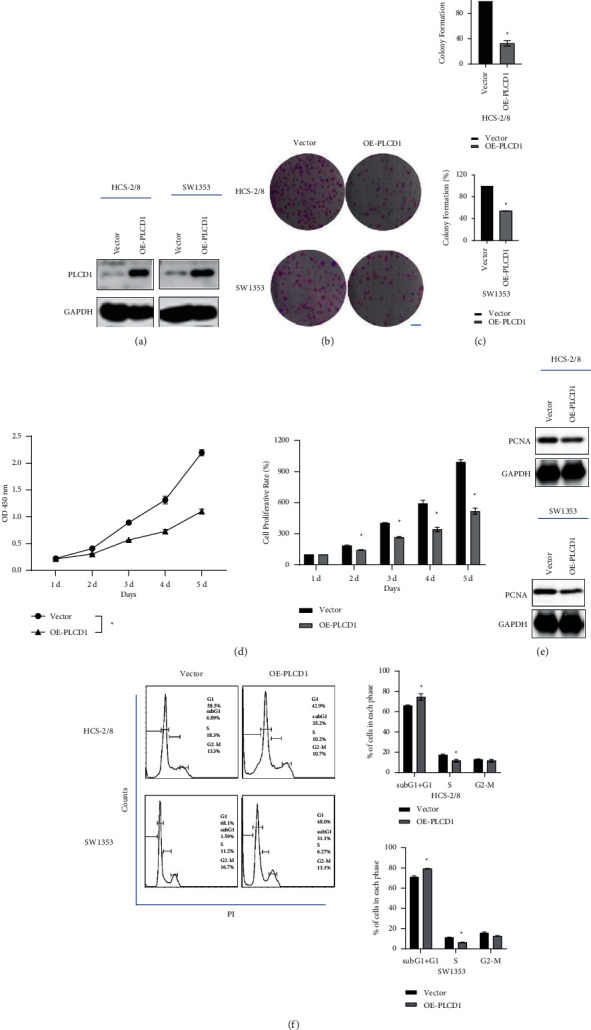
PLCD1 overexpression inhibited cell proliferation in vitro. (a) Establishment of OE-PLCD1 HCS-2/8 and SW1353 cell lines. (b, c) Colony formation of the OE-PLCD1 cell line. (d) CCK-8 assay kit revealed a downregulation of cell proliferation by PLCD1 overexpression. (e) Western blotting revealed a downregulation of PCNA expression by PLCD1 overexpression. (f) Flow cytometry analysis revealed cell cycle blocking by PLCD1 overexpression.

PLCD1 overexpression cell lines (OE-PLCD1 HCS-2/8 and OE-PLCD1 SW1353) were established based on each cell line (Figure 3(a)). The colony number decreased in OE-PLCD1 cell lines, and the colony formation rate of OE-PLCD1 reduced by over 50% compared with the vector group (Figures 3(b) and 3(c)). CCK-8 assay revealed that cell viability decreased in the OE-PLCD1 group (Figure 3(d)). In addition, we also examined the PCNA expression in OE-PLCD1 cells by western blotting (Figure 3(e)), and PCNA expression was decreased by overexpressing PLCD1. Flow cytometry assay of cell cycle revealed a higher sub-G1 and G1 phase in OE-PLCD1 cells (Figure 3(f)). Our results demonstrated that PLCD1 overexpression in chondrosarcoma cells in vitro inhibits cell proliferation by arresting the cell cycle at the G1/S phase.

### 4.5. Cyclin-Dependent Kinases (CDKs) Expression Was Downregulated by Overexpressing PLCD1 In Vitro and In Vivo

**Figure 4:**
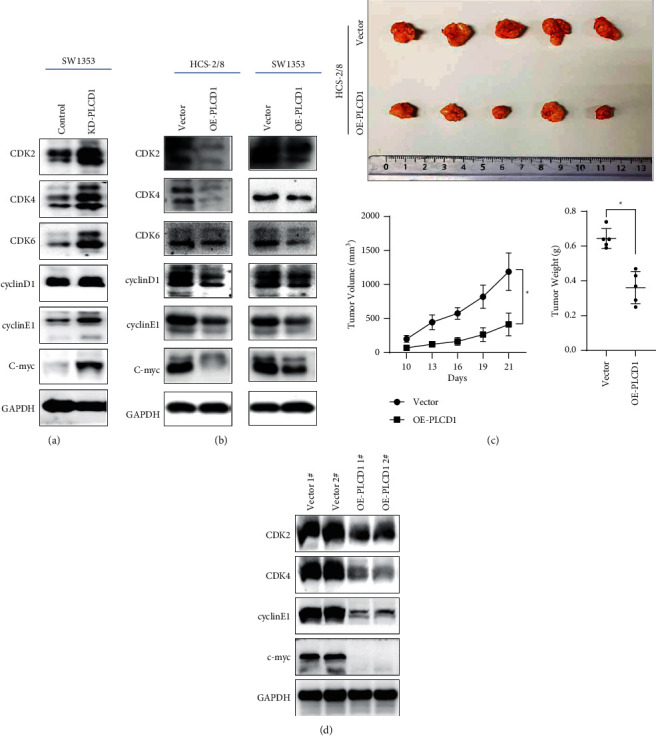
Cyclin-dependent kinases (CDKs) expression was downregulated by overexpressing PLCD1 in vitro and in vivo. (a) Western blotting of CDKs and cyclins in KD-PLCD1 cell line in vitro. (b) Western blotting of CDKs and cyclins in OE-PLCD1 cell line in vitro. (c) Antitumor effect of PLCD1 overexpression in vivo. (d) Western blotting of CDKs and cyclins in OE-PLCD1 cell line in vivo.

CDKs and their interacted cyclins were necessary for chondrosarcoma cell proliferation [21]. We further investigate the reason of cell proliferation inhibition by overexpressing PLCD1, and we hypothesized overexpressed PLCD1 impacted on CDKs and cyclins directly according to the above flow cytometry results. Western blotting revealed higher expression of CDKs and interacted cyclins in KD-PLCD1 SW1353 (Figure 4(a)) but opposite results in OE-PLCD1 SW1353 and OE-PLCD1 HCS-2/8 (Figure 4(b)). We further revealed the antitumor effect of PLCD1 overexpression in HCS-2/8 in vivo (Figure 4(c)), and Western blotting of the tumor tissue verified the downregulation of CDKs and interacted cyclins (Figure 4(d)). Our results demonstrated that overexpressed PLCD1 downregulated CDKs and interacted cyclins to prove a prolonged cell cycle in chondrosarcoma cells.

### 4.6. PLCD1-Induced DNA Damage and Caused Cell Apoptosis In Vitro and In Vivo

**Figure 5:**
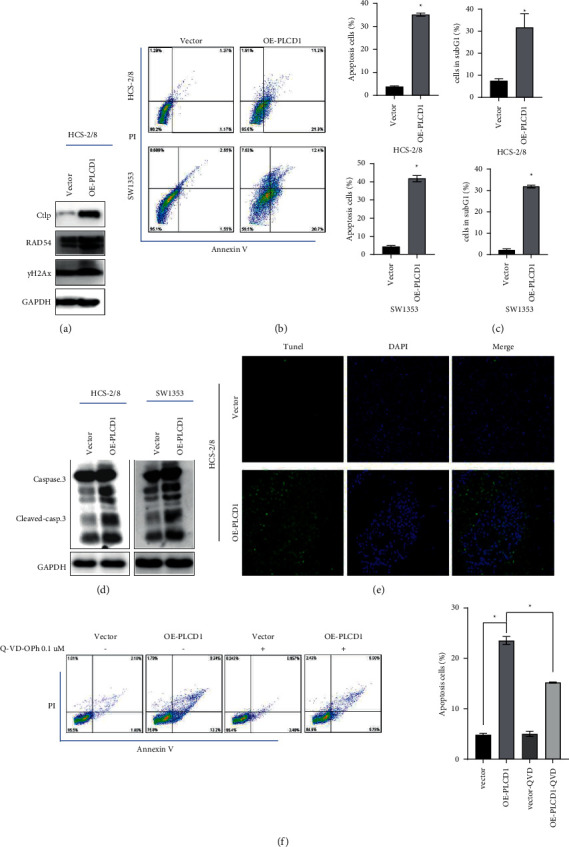
PLCD1-induced DNA damage caused cell apoptosis in vivo and in vitro. (a) Western blotting revealed DNA damage response protein upregulated by PLCD1 overexpression. (b) Flow cytometry analysis revealed cell apoptosis in OE-PLCD1 cell lines in vitro. (c) Significant increase of cells in the sub-G1 phase suggests a cell death population according to Figure 3(f). (d) Western blotting revealed cell apoptosis protein upregulated by PLCD1 overexpression. (e) TUNEL assay kit of OE-PLCD1 cell line in vivo. (f) Rescue experiment of cell apoptosis induced by PLCD1 overexpression.

Downregulated CDKs and interacted cyclins reflected a prolonged cell cycle but not the reason. We hypothesized PLCD1 overexpression induced DNA damage in chondrosarcoma cells. Western blotting revealed DNA damage response protein (CtIP and RAD54) had higher expression after PLCD1 overexpression (Figure 5(a)). Unrepaired DNA damage might cause cell death, and we further investigated cell apoptosis by PLCD1 overexpression. Flow cytometry analysis revealed that PLCD1 overexpression resulted in an increase of apoptotic cells in vitro, and a significant increase of cells in the sub-G1 phase suggests a cell death population by cell cycle analysis [19, 20] (Figures 5(b) and 5(c)). Western blotting verified caspase-3 and cleaved caspase-3 showed higher expression in OE-PLCD1 in vitro (Figure 5(d)), and immunofluorescence detected apoptotic cells colored by TUNEL staining in chondrosarcoma tissue in vivo (Figure 5(e)). To further demonstrate the contribution of PLCD1 on cell apoptosis regulation, the pharmacological caspase inhibitor Q-VD-Oph was applied to investigate the contribution of PLCD1 overexpression on cell apoptosis by flow cytometry (Figure 5(f)). These data show the inhibitor Q-VD-Oph significantly rescued cell apoptosis, induced by the PLCD1 overexpression. The above results indicated that PLCD1 promoted cell apoptosis and suppressed tumor cell proliferation in human chondrosarcoma cells, and our results demonstrated PLCD1 induced DNA damage and caused cell apoptosis in vitro and in vivo.

## 5. Discussion

The enrichment of cell proliferation-related genes is one of the typical features of chondrosarcoma. Our results demonstrate that PLCD1 overexpression can inhibit cell proliferation of chondrosarcoma. PLCD1 plays a key role in regulating both Ca^2+^ elevation and phosphoinositide metabolism [5]. Nakamura et al. have established PLCD1-deficient mice, which develop spontaneous skin tumors undergoing progressive hair loss [13]. Previous research shows the PLCD1 expression is downregulated in colorectal cancer cells [11], leading to induce E-cadherin expression, and PLCD1 overexpression reduced the malignant progression via E-cadherin and KRAS/MEK/ERK signal attenuation [9, 25–27]. Our results accord with the previous work and demonstrate that the inhibition of cell proliferation by overexpressing PLCD1 results from the downregulation of cell cycle-related protein, such as CDKs and interacted cyclins [23, 28].

We further find out the reason of PLCD1 blocking the cell cycle by analyzing the results of the flow cytometry. PLCD1 overexpression blocks the cell cycle at the G1/S phase, and PLCD1 knockdown has an opposite effect on the cell cycle of chondrosarcoma. We hypothesize PLCD1-induced DNA damage leads to the block of the cell cycle. Our results demonstrate that the upregulation of DNA damage response proteins suggests enhanced DNA repair occurring in PLCD1 overexpressed chondrosarcoma cells. In addition, cell apoptosis detected by flow cytometry analysis suggests the PLCD1 overexpressed chondrosarcoma cells failed to repair DNA damage and caused the programmed cell death [22, 29].

In conclusion, our study finds out that PLCD1 protein shows antitumor effect on chondrosarcoma via inhibiting cell proliferation. Overexpressed PLCD1 downregulates CDKs and interacted cyclins and induces DNA damage to cell apoptosis. PLCD1 may be a novel marker for the diagnosis of chondrosarcoma and classification of its malignancy. Drugs such as PLCD1 agonists might be useful for chondrosarcoma treatment. And PLCD1-induced DNA damage might enhance the chemotherapy sensitivity of chondrosarcoma. However, work is still moving forward for the reason that there still is no PLCD1 agonist now and PLCD1 deletion usually happens in tumor cells. A CRISPR-cas9 of the shRNA library is in need of further research.

## Data Availability

All data generated or analyzed during this study are included in this article.
